# Supplementary material for: SyNDI: synchronous network data integration framework
Source: BMC Bioinformatics. 2018 Nov 6;19:403. doi: 10.1186/s12859-018-2426-5 (PMC6219086; doi:10.1186/s12859-018-2426-5)
Supplement: Supplementary file 5 — Files of the example presented in Additional file 4. This additional file is a zip package that contains all files used in Additional file 4. Detailed descriptions of each of these files are presented in a README file included in this zip package. (GZ 30411 kb) [file 12859_2018_2426_MOESM5_ESM.gz › SyNDITools/Meme2Fimo/meme_4.9.0_4.tar.gz!/meme_4.9.0/doc/rna2meme.html]

rna2meme


# rna2meme

**Usage:** `rna2meme [options] <FASTA file>`

**Description:**

Convert an RNA sequence to its binding motif in MEME format.

**Input:**

FASTA file containing short RNA sequences.

**Options:**

-start <offset>
:   starting offset in rna sequence (inclusive); use negative numbers to count from end; default: 1

-end <offset>
:   ending offset in rna sequence (inclusive); use negative numbers to count from end; default: -1

-match <count>
:   count to assign to a match (complement); default: 1

-wobble <count>
:   count to assign to a U for a G, or a G for a U; default: 0.1

-miss <count>
:   count to assign to a non-match non-wobble; default: 0.01

-bg <background file>
:   file with background frequencies of letters; default: uniform background

-pseudo <total pseudocounts>
:   add <total pseudocounts> times letter; background to each frequency; default: 0

-logodds
:   output the log-odds (PSSM) and frequency (PSPM) motifs; default: PSPM motif only

-url <website>
:   website for the motif; The FASTA ID is substituted for MOTIF\_NAME; default: no url

**Output:**

Writes MEME format to standard output.
